# The Atmosphere in Relation to Disease

**Published:** 1855-12

**Authors:** J. A. Hingeston

**Affiliations:** Brighton.


### THE ATMOSPHERE IN RELATION TO DISEASE.

By J. A. Hingeston, M.R.C.S., Brighton.

That health depends upon changes of the weather, is a household aphorism, coeval with the earliest traditions. Hippocrates gave a large share of his attention to this circumstance; and his treatise on air, water, and places, is one of the choicest morsels of medical antiquity. At present, the inquiry is receiving an attention still more minute and exact than the physician of Cos had it in his power to bestow upon it. He knew nothing of the barometer and thermometer; of the composition of the sun's rays; of polarised light; of electricity and magnetism; or of the chemical mixture of the atmosphere with which we are surrounded, and which we breathe; and yet, considering how little he knew then, compared with what we know now, it is marvellous that he should have done so much with his scanty information, and that we should not do more with our intimate and increasing knowledge of the laws of nature. It would seem that science, whose data have the exactitude of mathematical demonstration, has rendered us idle in that faculty of the mind in which the ancients so particularly excelled; and has rendered us disdainful of those more vulgar qualities of hearing, sight, and touch, which constitute the united virtues of attention to, and the outward observation of, the outward phenomena of the world. Our fondness for intellectual abstractions confines us to our studios, instead of sending us forth into the open spaces of air and daylight for the purpose of *seeing* the multiform changes that are proceeding around us. The sight of the clouds that decorate the sky, and the varying aspect of the ocean, as capriciously beautiful as the clouds which it reflects, are not the sole property of the painter or the poet, but belong to the domain of science, and supply materials of study as profoundly philosophic as they are practically useful in unfolding the requisite conditions of life, health, and disease.

Every one, who has paid any attention to the subject, must be acquainted with the classification of clouds, viz., the cirrus, cumulus, and stratus, with their several subordinate combinations. It is not our present intention to enlarge upon these well known forms of vapour, but only to say a few words on one or two of their peculiarities relating to hygiene.

In saying that clouds are electrical machines, like Leyden jars, for instance, it is not meant to lay it down as an axiom that they are absolutely so, but merely to express what, upon the most attentive observation, they appear to be. They seem to be charged with positive and negative electricity, just like a glass rod excited by friction with silk. A negative and a positive pole seems to exist in each separate cloud—each being positively electric in its front, and negatively in its rear, or tail; and the small round cumuli, that follow each other successively on a clear fresh noon, are so many electric jars, or batteries, the positive front of the one being opposed to the negative tail of the other. It is not easy to *prove* that this is the fact: it is offered only as a fair presumption, and the present conclusion of the writer's mind. The proper motion of these clouds is rotary, each cloud revolving on its own axis, while the whole group or herd of them is proceeding forward with one common movement in the direction of the wind. It seems probable, that they are continually exchanging their electricities, one with another. If watched attentively, they will be seen to approach each other as it were by attraction, and then as suddenly to be repelled. But that which is incontestable about them is their circular movement.

After many years' observation, I am convinced that all clouds rotate on an axis of their own, the small as well as the large, the strati as well as the cumuli; and that this rotation is from left to right. The great tornados are known to be cyclonic, and so are the severe gales that blow up from the S.W., and sweep the agitated bosom of the Atlantic. The whirlwinds of sand in the desert, and the little whirls of sand that dance along the dusty road on a windy day, are instances, sometimes terrific, sometimes pleasing, of the rotary law of storms.

When these clouds are seen, the wind is from the west. They seldom, if ever, occur with a wind blowing from any other point of the compass. The electrometer is mostly negatively electric, or it is silent, or weak, The mortality is low, but illness is frequent, although of a curable and transient nature. Virulent epidemics do not prevail. The greatest epidemics that have passed over Europe between the years A.M. 1348 and A.D. 1850, have been accompanied with a wind blowing, with more or less force, from the N.W., E., or S.E., together with thick, heavy smelling fogs; swarms of locusts in the East, or small black flies in the West. The great plague, that was so many years travelling from China, A.M. 1348, was marked by an east wind, along with terrestrial commotions. The sweating sickness, A.D. 1551, was from the S.E. to N.W., and each time attended with a rainy, moist atmosphere. The influenza, A.D. 1729, passed over Europe from S.E. to N.W., with a moist, cloudy, foggy season; and the spotty fever of A.D. 1847, passed from E. to W. over the north of Europe, across Great Britain and Ireland, till it finally reached America in its westward progress. We have no means of knowing the electrical state of the air during the prevalence of these epidemics; but, judging by analogy, we should presume that the amount of electricity must have been very small, or none.

The electricity of the air and of the earth does not correspond. Sometimes the amount of electricity is aërial, while at other times it is telluric. Dark clouds are said to be negatively, and white clouds positively electric; always supposing they are not darkened by some accidental shadow. During the passage of white-headed cumuli, the electrometer is very active; in mists, the electricity is negative, feeble, or *nil:* but during the fall of rain, it is positive, and often very active. Sometimes the electrometer gives no sign of electricity, and yet the clouds may be exchanging their opposite electricities with each other, with the quickest vivacity. There may be incessant flashes of lightning, with or without thunder. At Brighton, on the night of 23rd of August, 1855, both sheet and zigzag lightning passed from cloud to cloud, without any audible thunder, and the electric gleams answered to each other from one extremity of the same cloud to the other, without ever descending to the earth. In this case, we beheld a kind of demonstration of the problem, that clouds are electrical machines in opposite states of electricity.

During "*thundery weather*" as it is called, head affections and bowel complaints are noticed. In highly sensitive constitutions, the coming on of a storm is presaged by vomiting, fainting, and a feeling of alarm. There is a peculiar symptom analogous to that which arises in cases of injury to the spine, viz., the colon being greatly surcharged with wind: it is an evidence of direct exhaustion of the nervous centres. When this state of atmosphere is prolonged, biliary congestion ensues, or, speaking more generally, venous congestions take place in the head, the chest, or the abdomen.

The large, white-headed cumuli that collect in clear bright days, and look so much like snow-capped mountains in the distance, are rotatory storms of hail, rain, or thunder, gyrating from left to right. If attentively watched for some time, this circular movement may be clearly perceived. The central void of this gyration of cloud is a calm, while the wind is blowing briskly or sharply round in the direction indicated. The hail or rain falls in the round of the circle, and sometimes lightning flashes from the opposite sides of its diameter. During the passage of one of these storms along the horizon of the sea, I have seen the lightning drop from one of its extremities into the ocean, and at the same moment a counter-stroke ascend from the sea to the cloud at its opposite extremity. In the intermediate space, beneath a magnificent cupola of clouds, a brilliant corruscation of electricity darts both ways at once, connecting the negative with the positive pole of the voltaic pile, in a twinkling. But, not only do these clouds form gyrating storms in themselves, whirling from left to right, but they are also moving, together with each other, in mass straightforward in the direction of the wind, which, when these clouds appear, is generally from S.S.W., the direction of the storm being N.N.E., although this is not invariably the case. At the same time, in distant and opposite quarters of the horizon, other masses of the same sort of cumuli may be observed, all of them distinct and wide apart from each other, the intervening portions of the sky being singularly clear and blue. Indeed, the whole prospect of the sea or land is very distinct, vividly coloured, and the outline of the distances beautifully fine. Several of these gyratory storms keep marching onwards in alternate spaces, marshalled in a vast circular array, and rolling round a circumference comprising an area of some fifty or a hundred miles or more, the centre of this circumference being a bright translucent calm. Thus, this vast gyration is composed of many rotatory storms moving *en échelon*, in grand round.

In the midst of the cold weather of the spring of 1852, the sultriness on the 22nd and 23rd of March was probably caused by our being suddenly in the centre of one of these cyclones; for the foreign news informed us that about this very time, an unexpected tempest swept the Adriatic, and did great mischief. In this case, the Gulf of Venice felt the circumference of the storm, of which we formed the centre. The westerly gales which blow up from the Atlantic, with so much violence, are very extensive rotatory storms, that advance up the channel with a right shoulder movement from west to east.

On the approach of one of these masses of vapour, the mercury, both in the barometer and thermometer, first falls and then rises, with great rapidity. For the most part the barometer stands at 29·500 inches: although nautical men are practically acquainted with the fact, that as the storm is collecting the barometer rises very considerably. Thus, it will go up as high as 30·370 inches, and then suddenly drop down to 29·170 or 28·900 inches, in the course of twenty-four hours or so. This statement is made from the records of direct observation. The mean height of the thermometer varies with the season of the year; but there is a sense of heat and oppression on the approach of these storms, and of a negative feeling of chilliness as they pass away. They chiefly appear in the spring, autumn, or midsummer.

The accompanying and residual state of the atmosphere is congenial to health. Vegetation freshens, animals are brisk, the cocks crow, and the swallows, those blithe associates of a summer day's ramble, skim along the lawn or the pond with the most delightful alacrity. These are the days when the garden looks and smells the sweetest, and the foliage is at its best. All nature revives; and the air we breathe at these moments is antagonistic of disease. Cases of debility experience a favourable reaction, and even the moribund reclaim a few short delusive intervals from the inevitable collapse of death.

One of the causes which may influence the nature and growth of the present generation of man, is certainly the predominance of a town life, which excludes both the enjoyment and benefit of fresh air and sunshine; and that the stature of the population is below the average height, common observation can assure us, without the indisputable evidence of statistics. Shut up within close apartments, removed from the direct rays of the sun, hidden from the sight of the blue sky and the white clouds, and immured beneath a canopy of smoke and lofty buildings, how is it possible that the functions of life can develope themselves at large, with their natural energy, and in their due proportions? It is evidently impossible; intra and extra-uterine vitality are equally arrested and deformed; the blood loses its full measure of oxygen, and is deprived of its ruby tint, so characteristic of health and vigour; the limbs are small, the joints large, the chest narrow, the forehead hydrocephalic, the teeth irregular, the eye wan, the hair lank, the mind morbidly keen, and the passions perverted or depraved. The happiness of the mountaineer is a felicity utterly unknown to the denizens of the great metropolis, the artisan, the clerk, the menial, the pauper, and those more exalted mortals who but half exist in the courts of law, the counting-house, the midnight dust of the House of Commons, or the brighter purlieus of Downing Street and St. James's. No fame, however laudable, can equal one hour spent in watching the morning break along the ocean, or in inhaling the fragrant breeze that whistles across the plain in the noontide hour.

But to return:—The vertical height of these large cumuli is very great. I once triangulated the depth of one that gathered over London several years ago, and thought I had reason to conclude that it was three miles deep; which, if I was correct, would give a body of vapour almost as high as the top of Mont Blanc. Arago, in his Meteorological Essays, just published, by Colonel Sabine, mentions that the vertical height of the clouds in which thunder and lightning are produced, was found by De l'Isle, member of the Academy of Sciences, to attain the enormous elevation of 8,080 metres, or 26,510 English feet!—a calculation which I believe is by no means exaggerated. Peytier and Hossard, two French engineers, stationed on the Pyrenees, found the thickness of some *ordinary* clouds more than half a mile (Brocklesby's *Meteorology*, New York, 1851); but the vertical height of these large cumuli is very considerably more than this. They are also very dense; and their density, coupled with their vast height, accounts for the darkness during their transit.

Arago, in the same Essay just mentioned, says that Captain Hossard had remarked, that there take place suddenly, at several points of the lower stratum of clouds, upward rushings, extending vertically like rockets—a precursive sign that does not appear to have been mentioned by any previous meteorologist. Nothing can be more exact than this description of a storm-cloud in process of formation. As far back as 1835, I had observed the same thing, at Hurst, in Sussex, while watching a thunder cloud forming above the downs facing me. A centre, or core of vapour descends to the ground vertically beneath the upward rushing, and forms the pivot on which the storm rotates. The upward rushing above, and the pivot below, seem to be produced by a vacuum of heated air in the centre of the mass. The pivot on the ground is grey, but the upward rushing is as white as wool, and as dense as a volume of smoke from the explosion of a powder magazine. Beccaria, the Italian meteorologist, observed and described the dark central tint that touches the ground, connecting it with the vapour above.

Another peculiarity of these large cumuli takes place just before they break up and dissolve. A kind of table-land, of white or yellowish white amorphous vapour, stretches itself high up in the sky above the peaks of the cloud, while, at the same time, below the white headed summits, a sheet of neutral tint is drawn horizontally athwart the mass. Then this neutral tint expanse breaks forth here and there into long dark strati, which, after a time, soften and melt away in rain. As the rain is falling, arms are thrown out from the neutral tint expanse; they approach, almost touch, and then repel each other. The arms turn away, are twisted up or down, and dissolve in floods of wet. It is an electric action: opposite electricities are exchanged, and most likely an explosion, more or less loud, ensues, just as the vapour is being condensed into water.

The entire atmosphere changes, and the sky, but now so clear and resplendent with majestic cumuli, is obscured by a low scud. Everything is dull and grey. Beneath the scud ragged fragments chase each other in horizontal lines several miles in length. There are usually two or three lines running parallel one above another; but at last they approach and coalesce. The hurry with which these fragments speed along is not a little remarkable; and not less so, is the manner in which they suddenly halt, reverse their action, meet, mingle together, and vanish from the sight.

It is in damp weather that dyspepsia chiefly prevails — the acid indigestion of gouty habits and valetudinarians, the scrofulous, the indolent, and the pitiable host of "*never-wells*." Exercise and manual labour in the open air is the legitimate panacea. The skin suffers from cold and moisture, which active exertion alone can obviate; and the bronchial tubes suffer along with the skin. This is the primary condition of rheumatism, which some have supposed to arise from a sudden loss of electricity, and have proposed, as a cure for the disease, insulating the patient by letting him sleep on a bedstead with glass legs, like the insulated stool of an electric machine. This supposition is so far true, that rheumatism occurs in damp weather when the amount of animal electricity is the smallest, and the most readily parted with.

Another peculiarity of the large cumuli is that, instead of breaking up and dispersing as the day declines, they halt, and one of them settles against the setting sun, who sends out his beams from behind its shadow, and tips its edges with gold. As the rays dart upward into the evening sky, they appear all the brighter from being contrasted with the dark mass in front of them. They are called "*Moses' horns*," and indicate rain, by showing how much the air is surcharged with vapour held in solution.

The sound of rain may be heard a long way off, even though the nimbus from which it is falling be out of sight. It may be heard out at sea by a person listening on the shore, or inland, as it rustles across the fields and copses. Elijah bade Ahab to make haste, because of the sound of an abundance of rain. Before rain, the sea makes a noise, and scoops the beach into hollows.

In the spring of the year, when the N.E. wind has been blowing for a long time, with a cloudless sky, a small darkish cloudlet forms in the eye of the S.W., and remains stationary there for some hours every day. This little cloud (I am writing from Brighton) is the forerunner of a gale from the Atlantic. It is most likely the focus or axis of a gyration of wind at the point where the N.E. meets the gale from the S.W., forming a condensed nucleus of vapour at this spot. It is the same as the small white cloud that presages the typhoon in the China seas, which is a cyclone of terrific force and msgnitude, except that, judging from the difference of colour, the one in the China seas is positively, while this in ours is negatively, electric.

During the prevalence of cholera, the cirrus cloud is rare; but the cirro-strati, which occupy a lower stratum of the atmosphere, are frequent at noon, and accompany the sun for three or four hours in his meridian height. Occasionally, black smoky looking cumuli (negatively electric?) float up from the N.W. A calm prevails. Indolent cirro-cumuli lodge over the hills. The distance is dim, and a sticky vapour, charged with small black flies, pervades everything. The barometer stands obstinately at 30 inches, and the wind is from the N.W. or S.E. During the cholera of 1564, the wind was from the S.E. to N.W.; and so it was in 1832 and 1849.

In the first volume of his *Cosmos*, Humboldt directs our attention to the variation of the needle during the prevalence of thunderstorms. He calls them magnetic storms, announced by perturbations of the needle, even when no trace of their luminous manifestations are seen in the celestial vault. But we are not sure whether the seat of the disturbing cause is to be sought for in the atmosphere above, or in the earth beneath. Some observers have remarked that the declination of the needle is very great during the prevalence of the Asiatic cholera, and that it also corresponds with certain changes in the vegetable kingdom, as, for instance, the potato rot. These curious observations require very extensive confirmation, before they can be relied on as data in the history of disease; but there seems no reason to question the main fact, that electro-magnetic agency plays an important part in the production and propagation of maladies, both in the animal and vegetable kingdoms.

We have some facts to show cause why we should connect disease with the greater or less amount of electricity, signified by the electrometer. It would seem that, in the non-electric states of the air, diseases of a low type prevail. Thus, in the Registrar-General's return for the week ending July 14th, 1855, we find it stated, at p. 232, "weak positive electricity throughout the week"; and, on referring to the mortality of the same date, at p. 225, it is there recorded, that the chief deaths were from small-pox, hooping-cough, scarlatina, diarrhœa, and typhus. And thus, on the contrary, in the week ending September 8th, at p. 296, the electricity is stated to be "positive", and the mortality, at p. 289, to be "not high for the season". During the prevalence of Asiatic cholera, the electricity is weak, or nothing: thus, in the week ending September 16th, 1854, on the 13th and 14th September, when cholera was at its maximum, the electricity, p. 377, is stated, "none was shown." Thus, again, for the week ending September 22nd, 1855, at p. 305, the mortality "shows a decrease of about 100 in each of the three previous weeks, and indicates a satisfactory state of the public health"; while, at p. 312, it is recorded that the electricity is "positive", "strongly positive", and "active throughout the day". Were we to connect health with positive electricity, as a settled thing, we might point out a curious connexion between the deaths of the young, and the continuance of a highly electric state of the atmosphere; but, as coincidence is not the same as cause and effect, we can only mention the isolated fact, that, in the week ending October 13th, 1855, it is stated, p. 333, that out of 870 deaths (or 225 below the average), 449 (or about one-half) were in persons under 20 years of age: the electricity (p. 340), being both positive and strong, as it had been for several weeks past. It would be pushing the facts too far, and laying ourselves open to the imputation of forcing a favourite theory to suit a particular purpose, were we to enlarge the number of our examples; but they are endless, and we disclaim any theory whatever. Let the medical inquirer make his own references, and judge for himself. Does positive electricity, long continued, predispose to inflammatory ailments? For, in the week ending October 27th, 1855, the electricity being, as it had been, both strong and positive, we find, p. 349, three cases of peritonitis particularly reported.

If we consider that every living creature is as much an electrical machine as each cloud; that the earth itself is the largest and most powerful electrical machine of all; and that all things are always exchanging their electricities with each other; and, furthermore, that a strong electro-galvanic current passed from the nose to the tail of a living mouse, can kill it on the spot; that a simple electric stroke will abolish the life of a fly; and that lightning destroys myriads of insects, as well as some animals and human beings, at a single flash, it is past contradiction that electricity must be a grand actor in every form of life, whether of health or disease. If we take an electrometer, and pass a powerful stream of electricity into it from a large electrical machine in full play, the gold leaf within the electrometer is whirled round with violence, shivered into atoms, and sent flying in fragments to the inside surface of the glass, in desperate haste, to escape and distribute the excess of electrical fluid to the nearest non-electrical bodies. It is, in fact, a tornado within the bottle; like the tornados of the tropics, which are, most likely, nothing else than convulsive equalizations of unequal electricities on a gigantic scale. The violence of the winds, if not their directions, seem to be electro-magnetic. There are storms that disturb the magnet, or the electrometer, or both at once. And the partial rarefaction of the air by heat, and its condensation by cold, hitherto employed for explaining the force and current of the winds, are, most likely, only striking parts of terrestrial electro-magnetism. The tornado within the bottle is a practical exemplification of this supposition. Moreover, the sensorial effects of the electric fluid are proof paramount of its pathological energy. The tingling produced by a shock from an electrical machine in action, and the blindness, or loss of consciousness, or death, produced by lightning, exhibit the development of morbid phenomena too plainly to be mistaken. We have, therefore, every possible reason for regarding the kind of clouds as indications of the kind of atmosphere in relation to disease; and the various forms assumed by the vapours condensing or dissolving in the air may be considered, not only as picturesque beauties in the landscape we are occupied in watching or sketching, but also as criteria for judging of some of the most potent effects resulting from the operation of an experiment, silently and delicately performed upon the functions and sensations of animated beings. These signs only require reducing to some familiar characters, in order to render them practically serviceable; and then, when once recognized, they might be read off at a glance, and brought into daily use, as easily as the dial-plate of the electric wire, the gauge that indicates the steam pressure of a locomotive, or the minute hand of our watch in counting the pulse at a patient's wrist.

Brighton, December 1855.

---

